# First objective evaluation of taste sensitivity to 6-*n*-propylthiouracil (PROP), a paradigm gustatory stimulus in humans

**DOI:** 10.1038/srep40353

**Published:** 2017-01-11

**Authors:** Giorgia Sollai, Melania Melis, Danilo Pani, Piero Cosseddu, Ilenia Usai, Roberto Crnjar, Annalisa Bonfiglio, Iole Tomassini Barbarossa

**Affiliations:** 1 Department of Biomedical Sciences, University of Cagliari, Monserrato, CA, I 09042, Italy; 2 Department of Electrical and Electronic Engineering, University of Cagliari, Piazza d’Armi, Cagliari, CA, I 09123, Italy

## Abstract

Practical and reliable methods for the objective measure of taste function are critically important for studying eating behavior and taste function impairment. Here, we present direct measures of human gustatory response to a prototypical bitter compound, 6-n-propyltiouracil (PROP), obtained by electrophysiological recordings from the tongue of subjects who were classified for taster status and genotyped for the specific receptor gene (*TAS2R38*), and in which taste papilla density was determined. PROP stimulation evoked negative slow potentials that represent the summated depolarization of taste cells. Depolarization amplitude and rate were correlated with papilla density and perceived bitterness, and associated with taster status and *TAS2R38*. Our study provides a robust and generalizable research tool for the quantitative measure of peripheral taste function, which can greatly help to resolve controversial outcomes on the PROP phenotype role in taste perception and food preferences, and be potentially useful for evaluating nutritional status and health.

Taste acts as a final checkpoint for food acceptance or rejection behavior and enables individuals to distinguish nutrient-rich food from noxious substances^1^,^2^. Taste sensitivity greatly varies among individuals and can strongly influence food preferences, nutritional status and health^3^. It is not surprising, in fact, that the same kind of food can be perceived very differently by different individuals. This partly depends on social and cultural factors, but there is also an important genetic component^4^. Within this nutritional context, PROP tasting, which is a common genetic trait present in all population groups across the globe^5^, has gained, in the last decades, considerable attention as a paradigm of general taste perception and as an oral marker for food preferences and eating habits that ultimately impacts on nutritional status and health^3^. This role is based on data showing that PROP sensitivity is associated with variation in perception and preference for various oral stimuli including fats and high-fat/high-energy foods^6^,^7^,^8^,^9^, selection of fruits and vegetables^10^,^11^,^12^, body composition^13^,^14^, plasma antioxidant status^15^, and the colon cancer risk^16^,^17^,^18^. Other studies, however, do not confirm these associations by reporting inconsistent results^19^,^20^,^21^,^22^,^23^,^24^. Several studies have focused on identifying the factors that lead to these divergent conclusions^4^. One of the major issues is the difficulty to obtain an objective measure of taste sensitivity in humans. This is due, in part, to the fact that sensory analyses are carried out by a multitude of psychophysical approaches which, albeit of simple application, imply highly subjective evaluations and may then produce measurement errors that account for up to 20% of phenotypic variance^25^. Despite their limitations, the psychophysical testing procedures are actually the only methods in use as they can be simply and rapidly applied to large populations of individuals at a low cost and without causing discomfort or pain to the examined subject. Some measurements of the degree of activation of taste system, at both central and peripheral level, have been reported^26^,^27^,^28^. To evaluate activation at the central level, these studies use the functional Magnetic Resonance (f MR) which cannot be practically applied to large numbers of subjects^29^,^30^. Therefore, based on these considerations, it would be of great interest to design and validate a practical and reliable method to measure human taste responses objectively and non-invasively on a large population sample. In this work, we present direct measures of the gustatory system activation following PROP stimulation, by electrophysiological recordings from the human tongue (hereafter called ElectroTastegram, ETG), as the objective evaluation of subject’s PROP phenotypes. The ETGs were achieved by using a low cost, disposable, non invasive device which allowed a fast, objective, reliable, and precise determination of the individual PROP taste sensitivity.

Individuals can be classified as PROP non-tasters, medium-tasters or super-tasters using well-established screening and classification by psychophysical methods^3^,^31^,^32^,^33^,^34^,^35^,^36^. Non-tasters are subjects who detect PROP only at high concentrations, or not at all, medium-tasters are those who perceive PROP as moderately bitter, while super-tasters are subjects who experience intense bitter sensation from the compound, even at very low concentrations. It is known that PROP tasting is controlled by three polymorphisms (A49P, V262A, I296V) in the *TAS2R38* bitter-taste receptor gene^37^,^38^ which give rise to two common haplotypes: PAV, the dominant (taster) variant and AVI, the recessive (non-taster) one. PROP-taster individuals possess the PAV/PAV or PAV/AVI diplotype, whereas non-tasters are homozygous for the recessive haplotype (AVI/AVI). Rare haplotypes (AAV, AAI, PVI, and PAI) have also been observed^39^. PROP sensitivity is also associated with the density of fungiform papillae on the anterior surface of the tongue^32^,^40^,^41^,^42^,^43^,^44^. In this paper, we compare two parameters (voltage amplitude and depolarization rate) describing the waveform of ETGs with the intensity of perceived PROP bitterness, PROP taster status, *TAS2R38* genotypes, and the density of fungiform papillae of subjects.

## Results

### PROP genotyping and phenotyping

**Figure 1:**
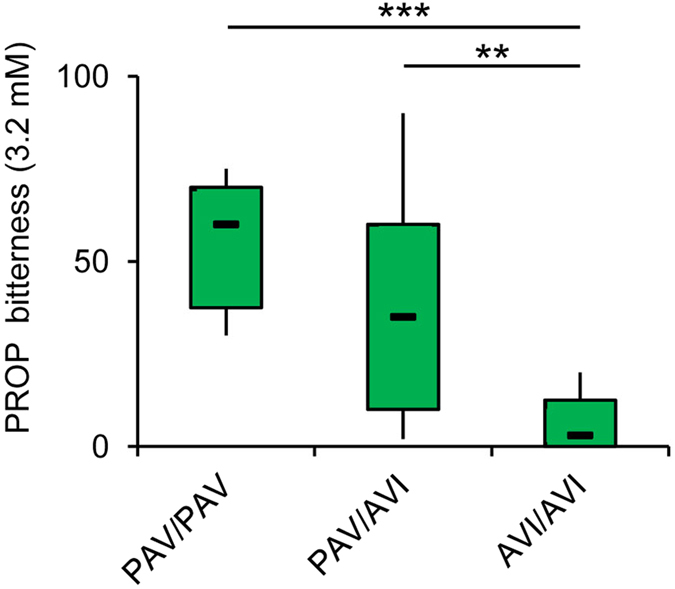
PROP bitterness intensity ratings (3.2 mM) according to *TAS2R38* polymorphisms. One-way ANOVA: F_2,40_ = 11.294; *P* = 0.00014. The box-and-whisker plots show the minimum, first quartile, median, third quartile, and maximum of each set of data. *n* = 7 individuals with genotypes PAV/PAV in *TAS2R38* locus, *n* = 25 PAV/AVI genotypes and *n* = 11 AVI/AVI genotypes. ***P < 0.001 determined by the Newman-Keuls post hoc test.

**Figure 2:**
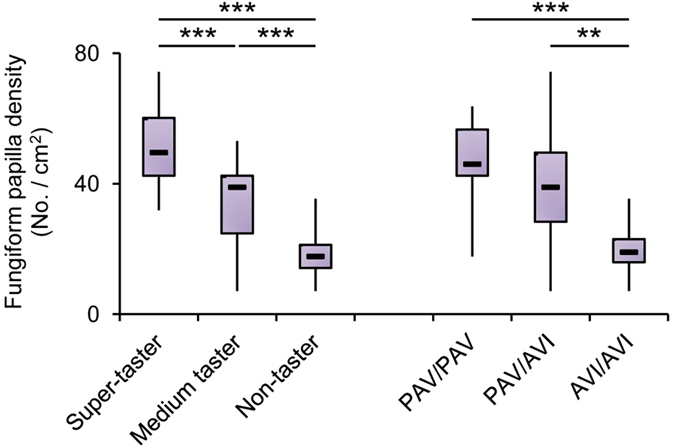
Relationship between the density of fungiform papillae and PROP taster status or *TAS2R38* polymorphisms. One-way ANOVA: F_2,40_ = 29.447; *P* < 0.00001 or F_2,40_ = 8.842; *P* = 0.0007). The box-and-whisker plots show the minimum, first quartile, median, third quartile, and maximum of each set of data. *n* = 15 super-tasters, *n* = 15 medium tasters and *n* = 13 non-tasters; *n* = 7 individuals with genotypes PAV/PAV in *TAS2R38* locus, *n* = 25 PAV/AVI genotypes and *n* = 11 AVI/AVI genotypes. **P < 0.01, ***P < 0.001 determined by the Newman-Keuls post hoc test.

Molecular analysis at the three single nucleotide polymorphisms (SNPs) of the *TAS2R38* locus identified 7 subjects who were PAV homozygous, 25 were heterozygous, and 11 were AVI homozygous. PROP bitterness intensity ratings (3.2 mM) were strongly associated with *TAS2R38* genotypes (*F*_2,40_ = 11.294; *P* = 0.00014) (Fig. 1). PROP bitterness ratings were lower in individuals with AVI/AVI genotype than in individuals with PAV/PAV or PAV/AVI genotype (*P* < 0.0016; Newman-Keuls test). No differences in bitterness intensity ratings between PAV/PAV and PAV/AVI subjects were found (*P* > 0.05). In addition, PROP taster groups differed statistically on the basis of their genotype distribution and haplotype frequency (*χ*^2^ > 50.00; *P* < 1.00e-008; Fisher’s test) (Table 1). Pairwise comparisons discriminated only non-tasters from the other groups on the basis of their genotype distribution and haplotype frequency (χ^2^ > 15.804; *P* < 0.00037; Fisher’s test). PROP super-taster subjects had a high frequency of PAV/PAV and PAV/AVI diplotypes, (40% and 60%, respectively), and PAV haplotype (70%), medium taster had a very high frequency of PAV/AVI diplotype (93.33%), whereas non-tasters had a very high frequency of AVI/AVI diplotype (84.62%) and AVI haplotype (92.31%). Density of fungiform papillae varied with PROP taster status or *TAS2R38* polymorphisms (*F*_2,40_ = 29.447; *P* < 0.00001 or *F*_2,40_ = 8.842; *P* = 0.0007) (Fig. 2). Super-tasters showed a higher density than medium taster, who had higher values than non-tasters (*P* < 0.0012; Newman-Keuls test). No differences in density of fungiform papillae were found between PAV/PAV or PAV/AVI subjects, and both showed values higher than subjects with AVI/AVI genotype (*P* < 0.004; Newman-Keuls test).

### Bioelectrical potentials and parameters defining PROP taste sensitivity

**Figure 3:**
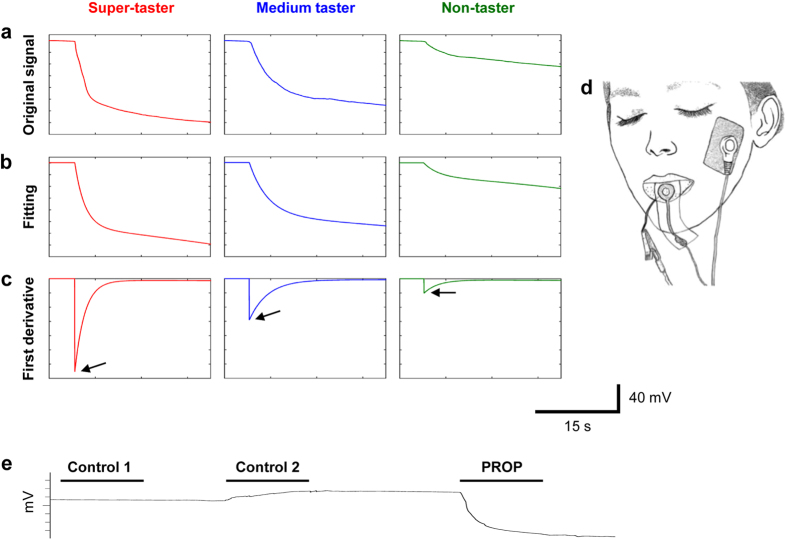
Examples of bio-electrical signals recorded from human tongue, and of waveform analysis. (**a**) The original signal of a representative super-taster, medium taster and non-taster. (**b**) The fitting of each original signal applied from the beginning of depolarization up to the next 15 s. (**c**) The first derivative calculated for each fitting. The maximum value of the first derivative, indicated by an arrow in each panel, was used as an index of depolarization rate of the original signal. The waveform analysis was performed for all participants (*n* = 43). (**d**) Drawing showing how electrodes were positioned for the differential electrophysiological recordings: one in contact with the ventral surface of the tongue, and one in perfect adhesion with the dorsal surface; a third disposable adhesive electrode (the ground terminal) was placed on the skin of the subject’s left cheek. This drawing shows that the electrode positioned on the dorsal surface of tongue leaves uncovered a circular area of the tip of the tongue, which was the same area where PROP stimulation was delivered during recordings and the density (No. of fungiform papillae/cm^2^) of fungiform papillae was calculated. (**e**) Response example to a sequence of three stimulations (15 s): paper disk (control 1), paper disk impregnated with 30 μl of spring water (control 2), and paper disk impregnated with 30 μl of PROP solution.

The differential electrophysiological recording between two silver electrodes, one in contact with the ventral surface of the human tongue and one in perfect adhesion with its dorsal surface, allowed us to measure bioelectrical potential variations in response to PROP bitter taste stimulation (Fig. 3). The waveform analysis of the recorded signals showed that PROP stimulation evoked negative monophasic potentials, characterized by a fast initial depolarization followed by a slow decline, which continued for the entire duration of the stimulation, and that the depolarization amplitude of signals, as well as the depolarization rate, were highly variable among subjects. The amplitude values varied from 5.8 mV (measured in a non-taster subject) to 138.3 mV (determined in a super-taster subject). An example of these differences is shown in Fig. 3, where the original signals of a representative super-taster, medium taster and non-taster are presented in Fig. 3a, while the fitting of each original signal applied (by using the formula: *f*(*t*) = *ae*^*bt*^ + *ce*^*dt*^) from the beginning of the depolarization up to the next 15 s, is in Fig. 3b. It is noteworthy that the maximum value of the first derivative (indicated by arrows in Fig. 3c), corresponding to 130, 56 and 21 mV/s in the super-taster, medium taster and non-taster respectively, was a representative index of the depolarization rate of signals. The typical negative monophasic potential variation that we found in response to PROP stimulation was not evoked by the two control stimulations (Fig. 3e). Control 1 (dry paper disk) produced no potential changes, while Control 2 (paper disk impregnated with spring water) evoked a small positive potential variation, probably due to the presence of electrolytes in water.

**Figure 4:**
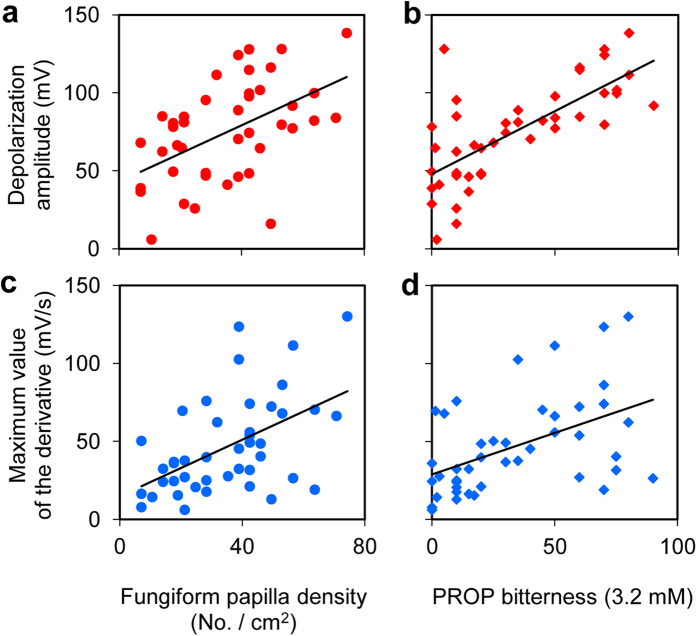
Linear correlation analysis between the parameters describing the waveform of signals and those defining PROP taste sensitivity. (**a**) Relationship between depolarization amplitude and density of fungiform papillae (*r* = 0.504; *P* = 0.00057). (**b**) Relationship between depolarization amplitude and intensity of perceived PROP bitterness (*r* = 0.741; *P* < 0.00001). (**c**) Relationship between the maximum value of the first derivative and density of fungiform papillae (*r* = 0.518; *P* = 0.00037). (**d**) Relationship between the maximum value of the first derivative and intensity of perceived PROP bitterness (*r* = 0.511; *P* = 0.00053). *n* = 43 participants.

Figure 4 shows the scatterplots depicting the relationship between the parameters describing the waveform of signals evoked by PROP stimulation applied in the small circular area of the tongue surface (amplitude of depolarization and the maximum value of the first derivative), and parameters defining PROP phenotype (density of fungiform papillae determined in the same area of the tongue surface and the intensity of perceived PROP bitterness). The variability of signal amplitude and the maximum value of the first derivative among recordings is well evident in the Fig. 4, in which the parameters describing the waveform of signals are shown on the Y axis, while those defining PROP phenotype are on the X axis. The amplitude of signals was linearly correlated with density of fungiform papillae (*r* = 0.504; *P* = 0.00057) (Fig. 4a) and with intensity of perceived bitterness (*r* = 0.741; *P* < 0.00001) (Fig. 4b), with a voltage drop reaching a maximum value of 138.3 mV in a super-taster subject who was homozygous for the tasting variant of *TAS2R38*. Also the maximum value of the first derivative was linearly correlated with the same parameters defining PROP phenotype (maximum value of the first derivative vs. papilla density: *r* = 0.518; *P* = 0.00037 or vs. intensity of perceived bitterness: *r* = 0.511; *P* = 0.00053) (Fig. 4c,d).

**Figure 5:**
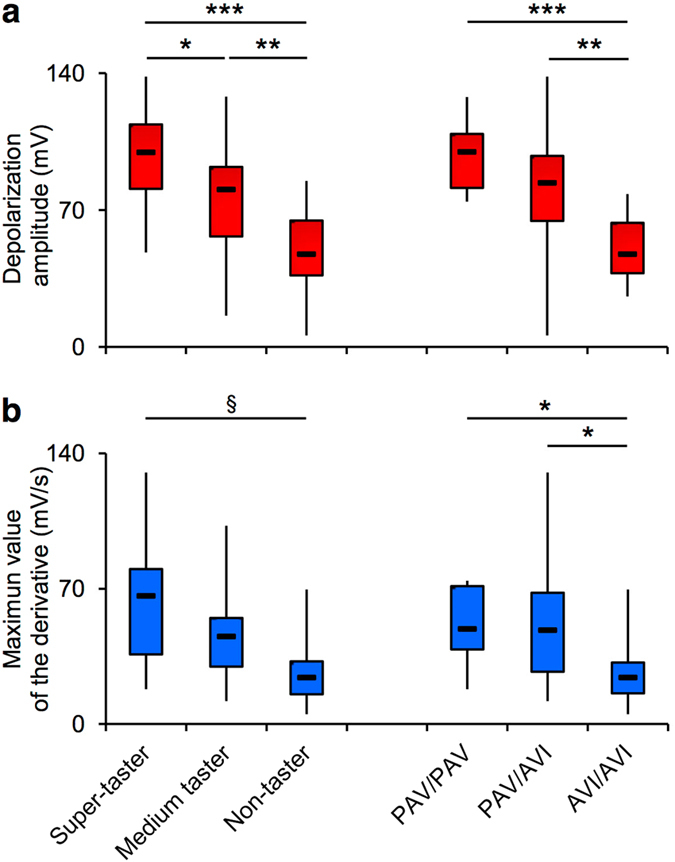
Relationship between the parameters describing the waveform of signals and the PROP taster status or *TAS2R38* polymorphisms. The box-and-whisker plots show the minimum, first quartile, median, third quartile, and maximum of each set of data. (**a**) Depolarization amplitude values. (**b**) The maximum value of the first derivative values. One-way ANOVA was used to analyze the effects of PROP taster status or *TAS2R38* polymorphism on depolarization amplitude and the maximum value of the first derivative (*F*_2,40_ > 3.699; *P* < 0.033). *n* = 15 super-tasters, *n* = 15 medium tasters and *n* = 13 non-tasters; *n* = 7 individuals with genotypes PAV/PAV in *TAS2R38* locus, *n* = 25 PAV/AVI genotypes and *n* = 11 AVI/AVI genotypes. *P < 0.05, **P < 0.01, ***P < 0.001 determined by the Newman-Keuls post hoc test; ^§^*P* < 0.001 determined with Duncan’s test since the assumption of homogeneity of variance was violated.

One-way ANOVA showed that the depolarization amplitude of signals, as well as the maximum value of the first derivative, varied with PROP taster status (amplitude: *F*_2,40_ = 12.836; *P* = 0.00005; maximum value of derivative: *F*_2,40_ = 7.6284; *P* = 0.00156) or *TAS2R38* diplotypes (amplitude: *F*_2,40_ = 7.552; *P* = 0.00165; maximum value of derivative: *F*_2,40_ = 3.699; *P* = 0.0333) (Fig. 5). Post-hoc comparisons showed that the values of signal amplitude determined in non-tasters were lower than those of medium tasters (*P* = 0.0072; Newman-Keuls test) who gave in turn lower values than super-tasters (*P* = 0.02776; Newman-Keuls test), and the values of AVI/AVI subjects were lower than those of PAV/PAV or PAV/AVI subjects (*P* < 0.0128; Newman-Keuls test) (Fig. 5a). In addition, post-hoc comparisons showed that the maximum values of the derivative determined in non-tasters or AVI/AVI subjects were lower than those of super-tasters (*P* = 0.00049; Duncan’s test) or subjects with at least one taster variant in *TAS2R38 (P* ≤ 0.0317; Newman-Keuls test), respectively (Fig. 5b).

**Figure 6:**
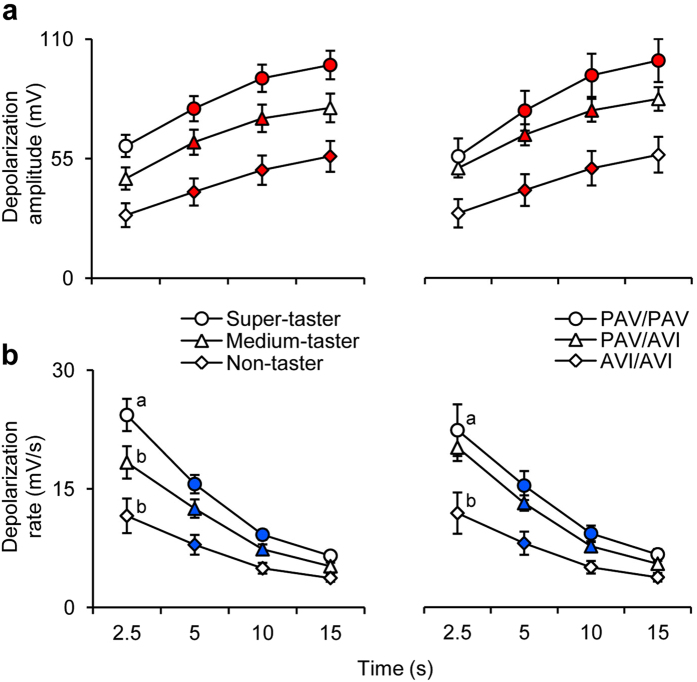
Time course of depolarization amplitude (**a**) or rate (mV/s) (**b**) across PROP taster or *TAS2R38* polymorphism groups during stimulation time. Data determined after 2.5, 5, 10, 15 s after application of PROP stimulation are shown as mean values ± s.e.m. Repeated measures ANOVA was used to compare values (depolarization amplitude across *TAS2R38* genotype of subjects: *F*_2.55,51.06_ = 3.030; *P* = 0.045; depolarization rate across taster status: *F*_2.24,44.79_ = 7.4814; *P* < 0.0011; or across *TAS2R38* dyplotypes: *F*_2.22,44.37_ = 3.6029; *P* = 0.031). *n* = 15 super-tasters, *n* = 15 medium tasters and *n* = 13 non-tasters; *n* = 7 individuals with genotypes PAV/PAV in *TAS2R38, n* = 25 PAV/AVI genotypes and *n* = 11 AVI/AVI genotypes. Different letters indicate significant difference (*P* ≤ 0.05; Newman-Keuls test). Solid symbols indicate significant difference with respect to the previous value of the corresponding group (*P* ≤ 0.021; Newman-Keuls test).

Mean values ± s.e.m. of signal amplitude, as well as depolarization rate (mV/s), determined after 2.5, 5, 10 and 15 s from the application of PROP stimulation according to PROP taster status and *TAS2R38* polymorphisms are shown in Fig. 6. Repeated measures ANOVA revealed that the time course of the depolarization amplitude showed a parallel shift toward proportionally increased values according to the taster group of subjects (*F*_2.5,50_ = 1.8388; *P* = 0.161) (Fig. 6a). On the contrary, the depolarization amplitude increased during the stimulation time as a function of *TAS2R38* genotype of subjects (*F*_2.55,51.06_ = 3.030; *P* = 0.045) (Fig. 6a). In fact, values significantly increased up to 15 s in homozygous PAV/PAV subjects (*P* ≤ 0.021; Newman-Keuls test), while only up to 10 s in the heterozygous and AVI/AVI subjects (*P* ≤ 0.0009; Newman-Keuls test). Repeated measures ANOVA showed that the depolarization rate, determined after 2.5, 5, 10 and 15 s from the application of PROP stimulation, depended on taster status (*F*_2.24,44.79_ = 7.4814; *P* = 0.0011) or *TAS2R38* dyplotypes (*F*_2.22,44.37_ = 3.6029; *P* = 0.031) (Fig. 6b). The trends of super tasters and medium tasters, or subjects with PAV/PAV and PAV/AVI genotypes, while being similar to each other respectively, differed significantly from those of non-tasters or AVI/AVI subjects. In particular, the depolarization rate values rapidly decreased down to 10 s in super-tasters and medium tasters (*P* ≤ 0.00022; Newman-Keuls test) or PAV/PAV and PAV/AVI subjects (*P* ≤ 0.0015; Newman-Keuls test), while only to 5 s in non-tasters (*P* = 0.0045; Newman-Keuls test) or AVI/AVI subjects (*P* = 0.021; Newman-Keuls test). In addition, the values determined in super-tasters at 2.5 s were higher than those of non-tasters (*P* = 0.0012; Newman-Keuls test) and of medium taster, although at the limit of statistical significance (*P* = 0.05; Newman-Keuls test), and those of PAV/PAV subjects were higher than those of AVI/AVI subjects, also in this case at the limit of significance (*P* = 0.05; Newman-Keuls test).

## Discussion

We found that ETG, used as a direct measure of the peripheral taste function in humans, is a highly reliable and yet moderately non-invasive method that allows us to obtain objective and quantitative data which are not affected by the individual’s subjective confounding factors. For the first time, since the discovery by Arthur L. Fox in 1932^45^ of the genetic sensitivity to the bitter taste of thiourea compounds (PTC/PROP), which is the most extensively studied example of individual taste variability, it was possible to obtain an objective determination of a subject’s PROP chemosensory phenotype, not just relying on what he/she verbally reports, but on the direct measurement of the degree of activation of stimulated taste cells.

We found that PROP bitter taste stimulation evokes negative monophasic potentials, which possibly represent the summated voltages resulting from the response of stimulated taste cells. Conversely, the two control stimulations (dry paper disk and paper disk impregnated with spring water) were ineffective in evoking this response. Although the morpho-functional organization of taste cells is different among sensory organs, the ETG seems to allow us to observe an electrical activity similar to that already recorded in other sensory organs^46^,^47^, such as the olfactory epithelium of vertebrates including humans, where the electrical activity (recorded in a so called electro-olfactogram) has been described as the summated generated potential of the population of stimulated olfactory receptor neurons^48^. The negative monophasic potentials that we recorded from the human tongue in response to bitterness of PROP, were characterized by a fast initial depolarization followed by a slow decline, which continued for the entire duration of the stimulation and, in most recordings, even longer. This extended activation could reflect the persistence over time of bitter taste, and its slow increase of intensity, compared to other taste qualities^49^,^50^,^51^. The direct and linear correlation that we found between the amplitude of depolarization evoked by PROP bitter taste stimulation and the density of the fungiform papillae measured in the same area of the tongue where PROP stimulation was delivered, is indicative of the fact that the recorded signals can effectively correspond to the summated response of stimulated taste cells. This seems to be confirmed also by the differences in signal amplitude among the PROP taster and *TAS2R38* categories, which exactly reflect the differences in papilla density among the same groups. On the other hand, the high variability in signal amplitude found within categories, mostly in medium tasters or PAV heterozygous, could be due to differences in the PAV-TAS2R38 expression, which have been correlated with individual differences in bitter sensory perception^52^. However, the magnitude of recorded signals could be affected by minor systematical errors as for instance variability in the contact electrode-tongue.

Our findings also indicate that ETG is a quantitative measure of a subject’s taste sensitivity, and the direct association that we found between parameters describing the signal waveform (depolarization amplitude and depolarization rate) and PROP genotype and phenotype further confirm this. Specifically, we recorded the largest amplitude values in super-tasters, the subjects with the highest responsiveness to PROP, the smallest amplitude values in non-tasters, who show little or no sensitivity to PROP, and intermediate amplitude values in subjects with moderate responsiveness (medium tasters). As regards the relationship between signal amplitude and *TAS2R38* gene, our results indicate that one variant with high affinity for PROP was already sufficient to cause high amplitude values. In fact, the values determined in PAV/PAV and PAV/AVI were higher than those of AVI/AVI subjects, who are homozygous for the non-tasting variant in the gene codifying the specific receptor TAS2R38. Moreover, the analysis of the time course of the response showed that the presence of two PAV alleles (as opposed to one) confers no additional advantage in the initial phase (2.5 s) of the response, which is certainly the most significant one to induce a behavioural response in a subject. These results are consistent with data showing that possessing a copy of PAV alleles gives no supplementary advantage for perceiving more bitterness intensity from PROP^53^. Our results instead seem to indicate that the presence of two PAV alleles is more important in the last portion of response. This is difficult to explain as one cannot exclude that the recorded potentials, in this part of the response, can be affected by electrical currents induced by lingual epithelial transport and/or related to salivary secretion.

On the other hand, the direct relationship we found between the signal depolarization rate and the parameters defining PROP genotype and phenotype could explain the shorter latency between stimulus application and the onset of perceived PROP bitterness which has been observed in super-tasters and medium tasters as compared to non-tasters^34^. These Authors showed that super-tasters and medium tasters or PAV/PAV and PAV/AVI subjects had values of latency of ~2.5 s, almost twice shorter than those of non-tasters or AVI/AVI subjects. We consistently found that values of depolarization rate measured at 2.5 s in super-tasters or PAV/PAV subjects were about twice as high as those of non-tasters or AVI/AVI subjects, respectively. Moreover, we found that the maximum values of the first derivative, that we used as an index of depolarization rate, determined in non-tasters, were lower than those of super-tasters, and those of AVI/AVI subjects were lower than those of subjects with at least one tasting variant in *TAS2R38* gene. This result, together with the higher bitterness intensity perceived by the same subjects, suggests that one tasting variant in the specific receptor is sufficient to elicit a prompt and more intense perception of bitterness by PROP.

In the literature, a similar approach has been previously reported^26^,^27^: by using a clever molded resin gustometer, the authors showed that the lingual surface potential (LSP) can be measured in humans and the application of increasing concentrations of salt causes LSP changes which correlate with human salt taste. However, while the data are consistent, the technique makes use of a sensor adhering to the anterior lingual surface by vacuum and this, apart from being difficult to manufacture and extremely uncomfortable for the subject, may evoke activation of other sensory receptors, such as mechanoceptors or nociceptors. Unlike that approach, our method gets rid of any other interfering signal. However, it is noteworthy that after stimulation, the stimulus (i.e. a drop of PROP solution in this case) is still on the tongue: hence the signal takes very long times (several minutes) to go back to the baseline, if no washing is done. Nevertheless, the simplicity and the practicality of manufacture and application of our device, which is equipped with disposable and non-invasive contacts, and its relatively low cost, make the use of our system preferable as more reliable, to psychophysical tests on a large population sample. On the other hand, the direct and linear correlation that we found between both the depolarization amplitude and rate, and the intensity of the perceived PROP bitterness during stimulation, indicate that our bio-electrical measurements are fully consistent with common human psychophysical observations.

In conclusion, the direct relationships that we found between the parameters describing the waveform of signals and those defining PROP phenotype indicate that ETG is a simple and reliable method for the quantitative measure of the activation of peripheral taste function which can be of great help to resolve controversial opinions on the role of PROP phenotype in taste perception, food preferences, and nutrition^4^. It is tempting to think that this objective technique, which puts together scientific rigor with methodological simplicity and moderate non-invasiveness, may be applied, also with other taste qualities (see Supplementary Fig. 1) (even in combination with psychophysical approaches thus extending the efficacy of the method), in studies aimed at evaluating eating behavior and taste function impairments in humans, or in different experimental models such as targeted knock out rodents and models of deranged feeding behavior.

## Methods

### Subjects

Forty-five non-smoking volunteers (17 males, 28 females, age 28.6 ± 0.86 years) were recruited according to standard procedures at the local University. No statistical methods were used to pre-determine sample size, but our sample size is similar to that generally employed in the field. All were Caucasian and were originally from Sardinia, Italy. They had a normal body mass index (BMI) ranging from 18.6 to 25.3 kg/m^2^. None were following a prescribed diet or taking medications that might interfere with taste perception. Subjects neither had food allergies, nor scored high on eating behavior scales (assessed by the Three-Factor Eating Questionnaire^54^). Thresholds for 4 basic tastes were evaluated in all subjects in order to rule out any gustatory impairment. All subjects were verbally informed about the procedure and the aim of the study. This trial was registered at ClinicalTrials.gov (identifier number: UNICADBSITB-1). Subjects reviewed and signed an informed consent form. The study was conformed to the standards set by the latest revision of Declaration of Helsinki and the procedures have been approved by the Ethical Committee of the University Hospital Company (AOU) of Cagliari, Italy.

### Study Design

Each subject (out of 45) was tested in two sessions in two consecutive days. For women, the sessions were scheduled around the sixth day of the menstrual cycle to avoid taste sensitivity changes due to the estrogen surge^55^. All subjects were requested to refrain from eating, drinking (except water), and using oral care products or chewing gum for at least 8 h prior to testing. They had to be in the test room 15 min before the beginning of the session (9.00 AM) in order to adapt to the constant environmental conditions (23–24 °C; 40–50% relative humidity).

Subjects were classified for their PROP taster status in the first session, while in the second session, they were assessed for the bioelectrical potential changes generated following PROP stimulation on a small circular area of the tip of tongue surface, and for the density of fungiform papillae of the same area of the tongue surface. Electrophysiological recordings were performed by observers who were blind to the *TAS2R38* genotype and PROP taster status of subjects.

All solutions (in spring water) used for the assessments were prepared the day before each session and stored in a refrigerator until 1 h before testing. Stimuli were presented at room temperature.

During the first visit, a sample (2 ml) of whole mixed saliva was collected from each subject into an acid-washed polypropylene test tube by means a soft plastic aspirator as it flowed into the anterior floor of the mouth. The samples were stored at −80 °C until molecular analyses were completed as described in the following section.

### Molecular analysis

Subjects were genotyped for three single nucleotide polymorphisms (SNPs) rs713598, rs1726866, rs10246939 respectively at base pairs 145 (C/G), 785 (C/T), and 886 (G/A) of the *TAS2R38* locus. The *TAS2R38* SNPs give rise to 3 non-synonymous coding exchanges: proline to alanine at residue 49; alanine to valine at residue 262; and valine to isoleucine at residue 296. These substitutions result in two major haplotypes (PAV, the dominant taster variant and AVI, the recessive non-taste variant) and three rare (AAI, PVI and AAV). DNA was extracted from saliva samples using the QIAamp^®^ DNA Mini Kit (QIAGEN S.r.l., Milano, Italy) according to the manufacturer’s instructions. Purified DNA concentration was estimated by measurements at an optical density of 260 nm. A polymerase chain reaction (PCR) was employed to amplify the short region of the *TAS2R38* gene including the first polymorphisms of interest (rs713598), followed by restriction analysis using *Hae*III, according to our previous works^40^.

For the rs1726866 and rs10246939 SNP of the *TAS2R38* locus the subjects were genotyped by using TaqMan^®^ SNP Genotyping Assay (C_9506827_10 was used for the rs1726866 assay and C_9506826_10 was used for the rs10246939 assay; Applied Biosystems by Life-Technologies Italia, Europe BV)^53^,^56^,^57^. PCR primers and TaqMan probes were designed by Applied Biosystems. The PCR tests were conducted in 96-well PCR plates with 10 μL in each well. Each reaction mixture contained 5 μL of TaqMan Genotyping Master Mix (codex 4371353, Applied Biosystems), 0.25 μL of the appropriate 40X Assay Primer/Probe Mix and a final concentration of 20 ng genomic DNA. The TaqMan PCR tests were carried out in an ABI 7000 sequence detector system (Applied Biosystems) under the following conditions: initial denaturation at 95 °C for 10 min, followed by 40 cycles of denaturation at 92 °C for 15 min, and annealing and extension at 60 °C for 1 min. After PCR tests were completed, the fluorescence of plates was read (60 °C for 1 min) in an ABI 7000 sequence detector system, and the results analyzed by allelic discrimination of the sequence detector software (Applied Biosystems). Replicates and positive and negative controls were included in all reactions.

### PROP Taster Status

In order to classify subjects for their PROP taster status, each volunteer was tested twice by using two different suprathreshold measures. Each subject was first assessed using the three-solution test according to Tepper *et al*.^31^, which has been validated in several studies^58^,^59^,^60^. Briefly, the taste intensity rating for three suprathreshold PROP (0.032, 0.32, and 3.2 mmol/L) (Sigma-Aldrich, Milan, Italy) and sodium chloride (NaCl; 0.01, 0.1, 1.0 mol/L) (Sigma-Aldrich, Milan, Italy) solutions was collected in each subject, by using the Labeled Magnitude Scale (LMS)^61^, which gave subjects the freedom to rate the intensity of PROP bitterness relative to the “strongest imaginable” oral stimulus ever experienced in their life. NaCl was used as a standard because taste intensity to NaCl does not change by PROP taster status^31^. Concentrations were tested (10 mL samples) in a random order. Subjects who gave lower intensity ratings to PROP solutions than those to NaCl were classified as PROP non-tasters, those who gave similar ratings to the two stimuli were classified as medium tasters, and those who gave higher ratings to PROP than to NaCl were classified as super-tasters. After a 1-hour period for recovering, the assignment of each subject to a PROP taster group (super-taster, medium taster, or non-taster) was validated using the impregnated paper screening test^33^,^36^. This method is based on the ratings of two 2 paper disks, one impregnated with NaCl (1.0 mol/l) and the other with PROP solution (50 mmol/1). Each subject was asked to place the paper disk with the taste stimulus on the tip of the tongue for 30 seconds or until the disk was thoroughly wet with saliva, and then spit it out. After tasting each paper disk, subjects placed a mark on the LMS scale (above describe) corresponding to their perception of the stimulus. Subjects were categorized as non-tasters if they rated the PROP disk lower than 13 mm on the LMS; they were categorized as super-tasters if they rated the PROP disk higher than 67 on the LMS. All others were classified as medium tasters^36^. In both methods subjects were instructed to rinse their mouth with spring water at room temperature before and between tasting any two solutions or paper disks; the interstimulus interval was 60 s. Two subjects classified as supertasters by using the three-solution test resulted as medium tasters when was used the impregnated paper screening test. These two subjects were excluded from other tests.

Since super-taster subjects could overestimate the oral stimuli, as compared to the other taster groups^3^, the subjects who resulted as super-tasters by using the LMS scale in the two suprathreshold measures were also trained by using the general labeled magnitude scale (gLMS)^62^, which expands the top anchor of the scale to include sensation of any kind. These subjects were asked to use the gLMS to rate the heaviness of 6 opaque, sand-filled jars ranging from 235 to 955 g, and heaviness ratings were used to normalize PROP taste intensity ratings as previously described^52^. These subjects resulted again as super-tasters.

Based on their taster group assignments, 13 of subjects were classified as non-taster (30.23%), 15 were medium taster (34.88%) and 15 were super-taster (34.88%). Three-way ANOVA was used to document the presence of the three taster groups (Supplemental Table 1).

### Electrophysiological recordings from the tongue

**Figure 7:**
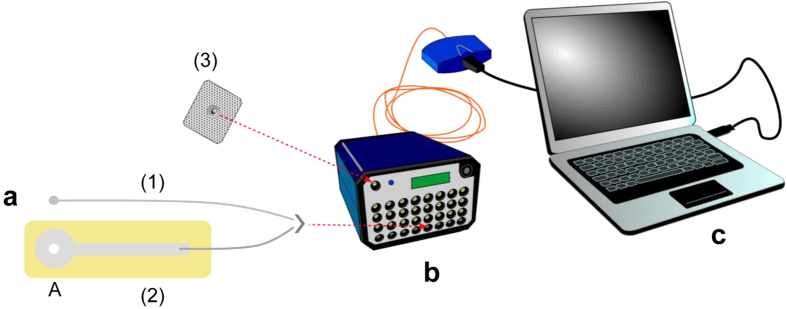
Layout of the measuring system used in the electrophysiological recordings. (**a**) Pair of electrodes used: electrode (1) was a silver wire (0.50 mm) with its distal end rolled up to form a ball (5 mm dia) in order to obtain good electrical contact and make the electrode safe with respect to the sublingual mucosa when positioned in contact with the ventral surface of the tongue; electrode (2) which was the main component of measuring system, was a silver film (100 nm thick) deposited on a thin (13 μm) polyimide layer (Kapton ©) (showed in yellow) by means of an evaporation technique in high vacuum; a film (2 μm thick) of insulating and biocompatible material (Parylene C) covered both sides of the electrode except for the circular area in its distal end (A), which represents the portion to bring in electrical contact with the tongue. This electrode has, in its circular end (A) a central hole (6 mm of diameter): when positioned in perfect adhesion to the dorsal surface of the tongue this hole leaves uncovered a round area of the tip of the tongue surface. In this area PROP stimulation was delivered during recordings and density (No. of fungiform papillae/cm^2^) of fungiform papillae was calculated. A third disposable electrode with adhesive solid hydrogel (CDES003545, Spes Medica, Italy) (3) was used as a ground terminal of the measuring instrument. (**b**) Scheme showing also the polygraph for human use employed (Porti7; TMSInternational, The Netherlands) with its USB to optic fiber adapter). (**c**) And PC for digitalization, recording and visualization in real time of bio-potential signals.

Differential electrophysiological recordings from the tongue were performed between two silver electrodes, one in contact with the ventral surface of the tongue and one in perfect adhesion with the dorsal surface. The layout of the measuring system (Italian patent pending N. 102016000057785) used in the electrophysiological derivations is shown in Fig. 7. One electrode (electrode 1, Fig. 7a) was a silver wire (0.50 mm) (WPI Sarasota, USA) with its distal end rolled up to form a small ball (5 mm dia) in order to obtain good electrical contact and make the electrode safe with respect to possible injury or irritation of the sublingual mucosa when positioned in contact with the ventral surface of the tongue. The second electrode (electrode 2, Fig. 7a) was the main component of the measuring system. It was made by depositing a silver film (100 nm thick) on a thin (13 μm) polyimide layer (Kapton©), by means of an evaporation technique in high vacuum. A film (2 μm thick) of insulating and biocompatible material (Parylene C) was deposited by Chemical Vapour Deposition (CVD) in vacuum, in order to cover both sides of the electrode except for the circular area (A) at its distal end, which represents the portion which must be in electrical contact with the tongue. This electrode was positioned in contact with the dorsal surface on the left side of the tip of the tongue taking advantage of its extreme thinness which allowed a conformal contact (i.e. a perfect adhesion) with the tongue surface after drying, with a piece of filter paper, the film of saliva normally present. At the center of area (A) there is a round hole (6 mm dia) which, after positioning, allowed free access to the tongue surface. This is where PROP stimulation was delivered during the electrophysiological recordings and where the density of fungiform papillae was calculated as described below. A third disposable electrode with adhesive solid hydrogel (CDES003545, Spes Medica, Italy) (3), like those adopted in current electrocardiographic practice, was placed in an electrically neutral position with respect to the phenomenon under observation (on the skin of the subject’s left cheek). This electrode was used as a ground terminal of the measuring instrument. The bio-potentials were bipolarly recorded with the Porti7 portable physiological measurement system (TMS International B.V., The Netherlands) (Fig. 7b), exploiting the AUX channels for a wider dynamic range (±3 V, rather than the 150 mV of standard bipolar ExG channels). The high input impedance of these channels, equal to 10^10^ Ω, ensures that the current drained from the subject is minimal, thus with very limited perturbation of the measured system. The analogue signals were sampled at 2048 Hz and digitized at 22 bits with 1.43 μV resolution, then sent to the host PC via an optical fiber connection (terminated with a USB adaptor) for real-time visualization, pre-processing and digital recording through the Polybench software (Fig. 7c). The Porti7 device is an isolated certified Class IIa medical device with type CF applied parts. For each subject, the recording started after establishing a stable baseline and lasted 60 s (15 s baseline, 15 s during PROP simulation and 30 s after stimulation).

Afterwards, the original signal was imported in Matlab (The MathWorks, Inc.) and filtered with a 86^th^ order linear phase Equiripple Finite Impulse Response (FIR) filter (cut off at 6 Hz) to reduce high frequency interference, thus extracting the global trend of the signal (depolarization) in a smoother, but still accurate, way. A curve fitting was applied from the beginning of depolarization up to the next 15 s by using a sum of two exponential functions, i.e. f(t) = ae^bt^ + ce^dt^ in the discrete time domain, and its first derivative was calculated. The curve fitting was applied to find the function from which to calculate the first derivative, as the first derivative computed on the original signal is affected by an excessive error due to the residual noise. The maximum absolute value of the first derivative was used as an index of depolarization rate. The biopotential waveforms were exported in ASCII format to be also analyzed by Clampfit 10.0 software, and their maximum amplitude value, as well as the values at 2.5, 5, 10 and 15 s, were determined. The values of depolarization rate (mV/s) at 2.5, 5, 10 and 15 s were then calculated.

### PROP taste stimulations

PROP taste stimulations were delivered by placing, for 15 s, a paper disk (6 mm dia) impregnated with 30 μl of (3.2 mM) PROP solution on the small circular area of tongue surface that was left uncovered by hole (A) in electrode 2. Each subject was instructed to rate the intensity of the PROP bitterness (3.2 mM) of the stimulation by LMS scale^61^.

In order to verify that the potential changes obtained following PROP stimulation were really due to PROP stimulation, a group of subjects (n = 30) were tested with the following sequence of stimulations (15 s): paper disk (control 1), paper disk impregnated with 30 μl of spring water (control 2), and paper disk impregnated with 30 μl of PROP solution. The interstimulus interval was at least 15 s.

### Density of fungiform papillae

Fungiform papillae density was measured according to Melis *et al*.^40^ and is briefly described as follows. During measurements, subjects sat on a chair, with elbows resting on a table, and supporting the head with the hands to minimize movement. The tip of the anterior tongue surface was dried with a filter paper and stained by placing (for 3 s) a piece of filter paper (circle 6 mm in diameter) that contained a blue food dye (E133, Modecor Italiana, Italy) on the left side of the tongue closest to the midline and spaced 4 mm from the edge of the tongue. This shift, with respect to the area which provides reliable measurements of density of fungiform papillae in high correlation with their total number on the tongue^44^, was imposed by the need to keep the stained circular area of the tip of the tongue perfectly overlapping with the same area where PROP stimulation was delivered during the electrophysiological recordings.

Multiple photographs of the stained area were taken of each subject using a Canon EOS D400 (10 megapixels) camera with lens EFS 55–250 mm. The digital images were downloaded to a computer and analyzed using a “zoom” option in the Adobe Photoshop 7.0 program. The fungiform papillae were identified and counted for each subject according to previously established criteria^40^,^44^. The density/cm^2^ was calculated. Papillae were separately identified and counted by three trained observers who were blind to the PROP status of subjects. The final measurements were based on the consensus assessment by all observers.

### Statistical analyses

Fisher’s method (Genopop software version 4.0; http://Kimura.univmontp2fr/~rousset/Genepop.htm)^63^ was used to test *TAS2R38* genotype distribution and haplotype frequencies according to PROP status. The relationships between the amplitude of signals, as well as the maximum value of the first derivative, and density of fungiform papillae, or the intensity of perceived PROP bitterness were assessed using linear correlation analysis. One-way ANOVA was used across PROP taster groups or *TAS2R38* genotype groups to compare mean values ± s.e.m. of fungiform papilla density, amplitude of biopotentials and the maximum value of the first derivative. One-way ANOVA was also used across *TAS2R38* genotype groups to compare mean values ± s.e.m. for PROP bitterness intensity ratings (3.2 mM). Repeated measures ANOVA was used across PROP taster groups or *TAS2R38* genotype groups to compare mean values ± s.e.m. of the amplitude of signals, as well as depolarization rate (mV/s), at 2.5, 5, 10, 15 s after the application of PROP stimulation. Data were checked for the assumptions of homogeneity of variance, normality and sphericity (when applicable). When the sphericity assumption was violated, a Greenhouse-Geisser correction or Huynh-Feldt correction was applied in order to modify the degrees of freedom. PROP taster groups or *TAS2R38* genotype groups matched for age and gender (data not shown). Post-hoc comparisons were conducted with the Newman-Keuls test, unless the assumption of homogeneity of variance was violated, in which case the Duncan’s test was used. Statistical analyses were conducted using STATISTICA for WINDOWS (version 7; StatSoft Inc, Tulsa, OK, USA). P values ≤ 0.05 were considered significant.

### Data availability

The data that support the findings of this study are available from the corresponding author upon request.

## Additional Information

**How to cite this article:** Sollai, G. *et al*. First objective evaluation of taste sensitivity to 6*-n-*propylthiouracil (PROP), a paradigm gustatory stimulus in humans. *Sci. Rep.* **7**, 40353; doi: 10.1038/srep40353 (2017).

**Publisher's note:** Springer Nature remains neutral with regard to jurisdictional claims in published maps and institutional affiliations.

## Supplementary Material

Supplementary Material

## Figures and Tables

**Table 1:** Genotype distribution and haplotype frequencies of *TAS2R38* SNPs according to PROP taster status.

	PROP status						*P*-value^1^
	Super-taster		Medium taster		Non-taster		
	*n*	%	*n*	*%*	*n*	*%*	
*Genotype*							
PAV/PAV	6	40.00	1	6.67	0	0	
PAV/AVI	9	60.00	14	93.33	2	15.38	<0.0001
AVI/AVI	0	0	0	0	11	84.62	
*Haplotype*							
PAV	21	70.00	16	53.33	2	7.69	<0.0001
AVI	9	30.00	14	46.67	24	92.31	

^1^*P*-value derived from Fisher’s method. *n* = 43.
