# Supplementary material for: First objective evaluation of taste sensitivity to 6-n-propylthiouracil (PROP), a paradigm gustatory stimulus in humans
Source: Sci Rep. 2017 Jan 11;7:40353. doi: 10.1038/srep40353 (PMC5225483; doi:10.1038/srep40353)
Supplement: Supplementary Material [file srep40353-s1.doc]

**Supplementary material for**

**First objective evaluation of taste sensitivity to 6-n-propylthiouracil (PROP), a paradigm gustatory stimulus in humans**

Giorgia Sollai^1+^, Melania Melis^1+^, Danilo Pani^2^, Piero Cosseddu^2^, Ilenia Usai^2^, Roberto Crnjar^1^, Annalisa Bonfiglio^2^, Iole Tomassini Barbarossa^1^*

^1^Department of Biomedical Sciences, University of Cagliari, Monserrato, CA, I 09042;

^2^Department of Electrical and Electronic Engineering, University of Cagliari, Piazza d'Armi, Cagliari, CA, I 09123 Italy

^+^These two authors contributed equally to this work.

**Corresponding author**: I. Tomassini Barbarossa, Department of Biomedical Sciences, Section of Physiology, S.P. 8, 09042 Monserrato, CA, Italy. E-mail: tomassin@unica.it; Telephone: +390706754144; Fax: +390706754191

Number of figures: 1

Number of tables: 1

**Supplementary Figure 1.** Examples of ETGs recorded in a subject in response to 30 µl of solutions representative of the five taste qualities.

**Supplementary Table 1.** Ratings of perceived taste intensity in response to three concentrations of PROP and NaCl in the taster groups.

|  | Super-taster  (*n* = 15) | Medium taster  (*n* = 15) | Non-tasters  (*n* = 13) |
| --- | --- | --- | --- |
| *PROP* |  |  |  |
| 0.032 mM | 6.08 ± 1.45 | 3.04 ± 0.69 | 0.89 ± 0.31 |
| 0.32 mM | 42.13 ± 2.88* | 34.33 ± 4.63 | 5.70 ± 1.98* |
| 3.2 mM | 84.45 ± 3.21* | 61.66 ± 5.00 | 22.08 ± 3.26* |
| *NaCl* |  |  |  |
| 0.01 M | 2.14 ± 0.67 | 2.02 ± 0.55 | 2.46 ± 1.33 |
| 0.1 M | 22.03 ± 3.11* | 23.4 ± 2.72 | 27.51 ± 3.23* |
| 1 M | 53.00 ± 5.09* | 62.36 ± 5.41 | 63.45 ± 3.65* |

Values are means ± s.e.m. *n* = 43. Three-way ANOVA was used to compare PROP intensity ratings with NaCl intensity ratings across groups (*F*_4,240_= 13.726; *P* < 0.00001).

* = significant difference between PROP and the corresponding NaCl concentration (*P* < 0.00017; Newman-Keuls test).
